# Supplementary material for: Impairment of IgG Fc functions promotes tumor progression and suppresses NK cell antitumor actions
Source: Commun Biol. 2022 Sep 14;5:960. doi: 10.1038/s42003-022-03931-7 (PMC9474879; doi:10.1038/s42003-022-03931-7)
Supplement: Supplementary file 2 — Supplementary Information [file 42003_2022_3931_MOESM2_ESM.pdf]

Supplementary Information:

Supplementary Figure S1: Association analysis between levels of tumor scIgGs and clinical prognostic factors of breast cancer patients.

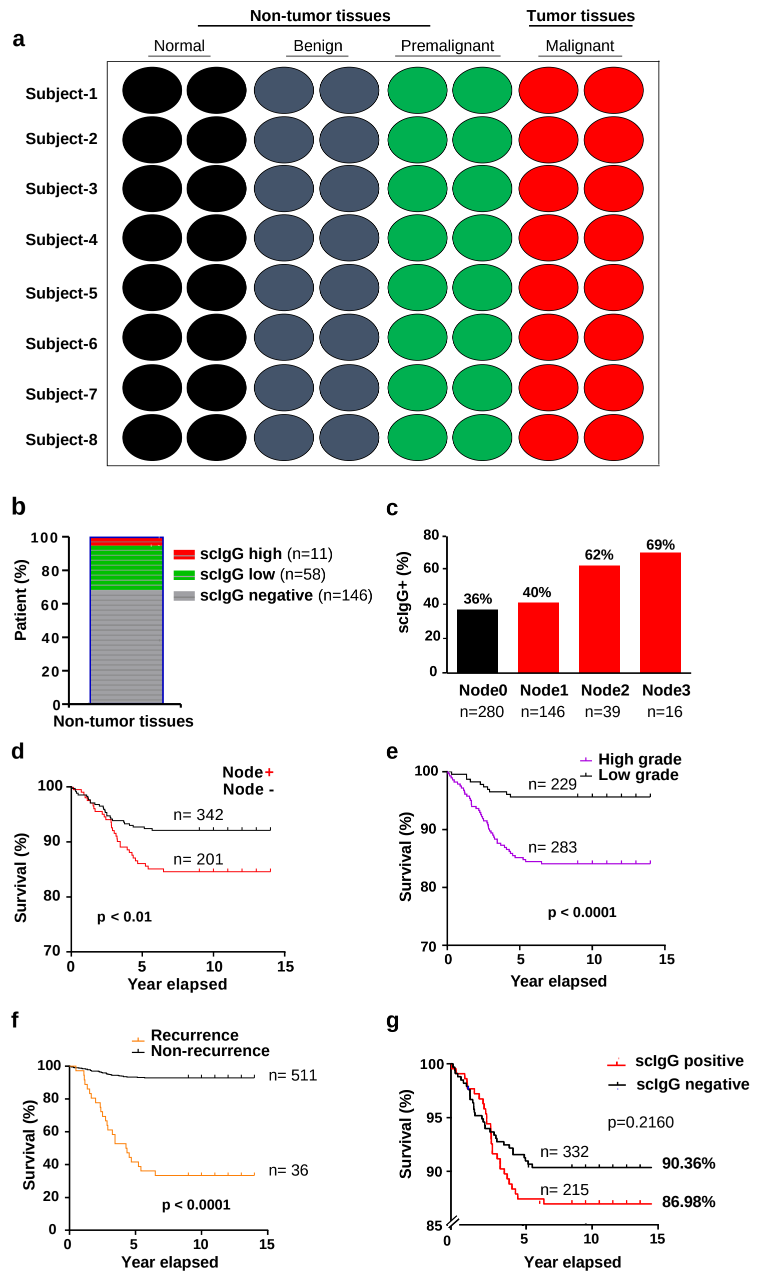

Figure S1: (**a**) Layout of TMA slides on glass slides used for scIgG detection using IHC staining method. Each slide contains 64 sample positions (8 subjects x 4 tissues types x 2 duplications). Non-

tumor tissues include adjacent normal (normal), benign tissue, premalignant, and tumor tissues (malignant). Duplicate tissues are indicated by the same colors. (**b**) Among patients (215) with scIgG-positive tumors, about 32% (Y-axis) of patients also had detection of scIgG stainings in non-tumor tissues, but only 5% patients was in scIgG-high (++/+++ IHC) levels. The same method and criteria used for tumor tissue staining were used to assess the scIgG staining intensity in non-tumor tissues. (**c**) Percentage of patients with scIgG positive staining in tumors is positively associated with lymph node metastasis in breast cancer patients. Node 1, 2, and 3 (X-axis) indicate increased lymph node metastasis and Node 0 is for no lymph node metastasis. Y-axis indicates percentages of patients with scIgG staining in tumor tissues in each group and cohort size in each group is indicated below the X-axis. (**d**) Patients with lymph node metastasis (node +, n=201) showed worse survival than those with node negative (node -, n=342) patients, p<0.01. (**e**) Patients with high grade tumors (n=283) had worse survival than those with low grade tumors (n=229). Tumor grade I is considered as low grade and grade II/III is considered as the high grade tumors, p<0.0001. (**f**) Patients with cancer recurrence (n=36) had worse survival rate than those without recurrence (n=511), p<0.0001. (**g**) Patients with positive scIgG staining in tumor tissues (n=215) had worse survival probability than those with negative scIgG staining in tumor (n=332), p=0.2160.

Supplementary Figure S2: Construction and validation of mouse tumor models**.**

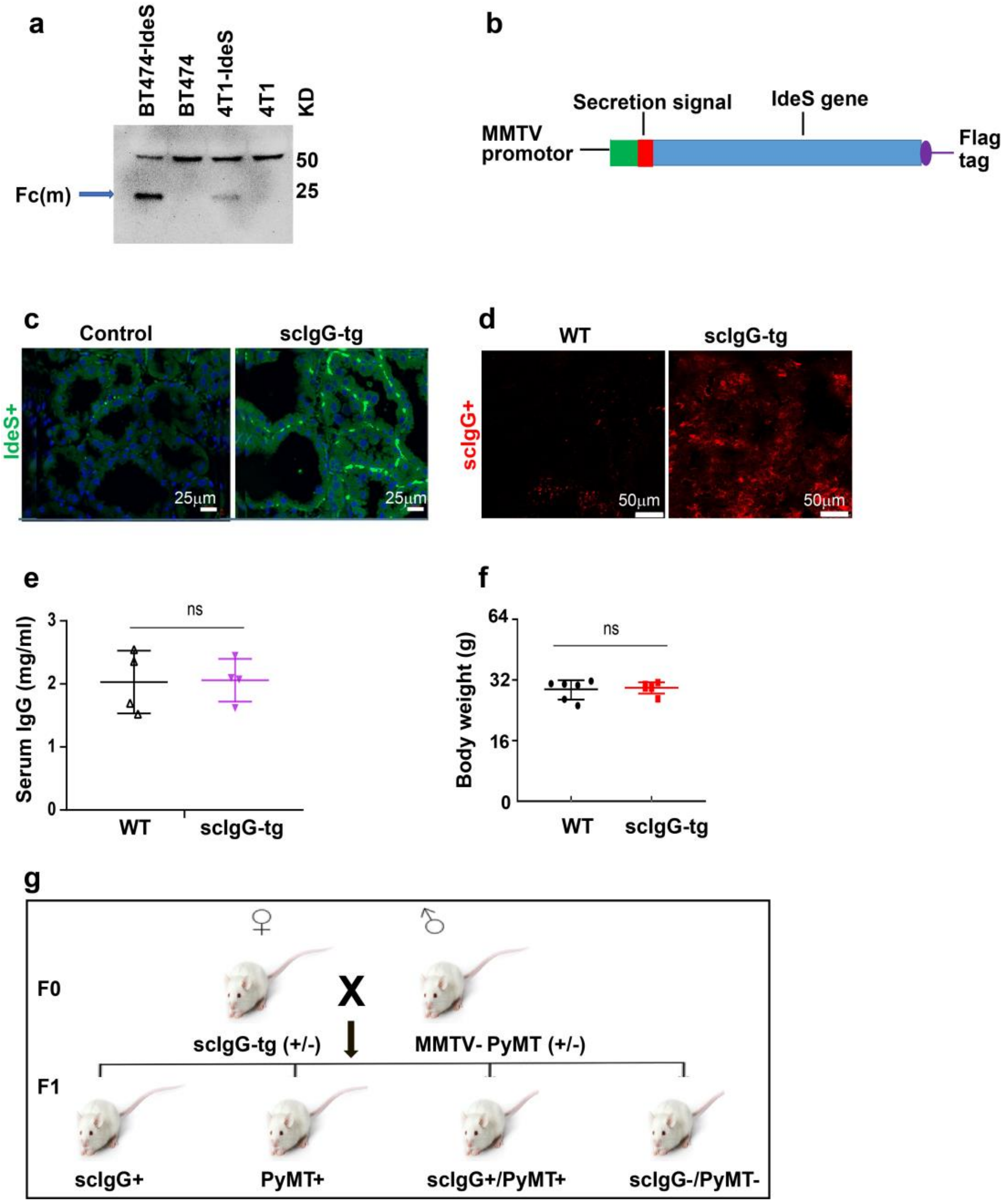

Figure S2. (**a**) Detection of hinge cleavage of scIgGs using Western blotting (WB) in BT474-IdeS and 4T1-IdeS cancer cells. Hinge cleavage scIgGs contain two different sizes of heavy chain: Fc(m) band (25KD) for a hinge cleaved Fc and full length heavy chain shows 50 KD molecular size. Cancer cells were incubated with mouse IgG3 (10μg/ml) in the cell culture media for 24 hours at 37°C. Total IgGs (both scIgGs and intact IgGs) in the culture media were enriched using protein A/G

beads and used for SDS-PAGE separation and WB detection using anti-mouse Fc specific antibody with HRP conjugation. (**b**) Diagram shows DNA construct used for generating scIgG+ transgenic mouse line. The DNA construct contains *IdeS* gene under MMTV promotor control and a Flag-tag at the c-terminus of the *IdeS* gene. (**c**) Expression of IdeS in mouse mammary tissue of IdeS expressing transgenic mice (scIgG-tg). Mammary tissues from female IdeS+ mice (scIgG-tg) and wild type age matched littermates (control) were sampled when the mice were at age of week 12 and 3 weeks after delivery of pups. The IdeS expression was assessed using immunofluorescence staining with a rabbit anti-Flag tag as primary antibody followed by an anti-rabbit antibody with FITC fluorophore conjugation. A representative image is shown for each group, n=3. (**d**) Detection of scIgGs in Met1 tumor tissues grown in scIgG-tg mice vs wild type (wt) FVB/n mice and a representative image for each tumor model (n=5) is shown. Murine Met1 tumor cells were implanted at mammary fat pads in age matched (6-8 week old) scIgG-tg mice and wild type FVB mice and tumors were preserved freshly in freezing media under liquid N2 at the end of *in vivo* study for IF staining. (**e**) Comparison of total serum IgG (mg/ml) levels in scIgG-tg mice and the counterpart wild type (wt) mice. There was no significant difference if serum IgGs between scIgG-tg and wt mouse group. Serum IgGs were measured using an ELISA kit according to the manufacturer's instruction, n=4. (**f**) The scIgG-tg mice showed no change in body weights in comparison with the wild type (wt) age matched littermate mice. The mice body weight were weighted at the same mature age for scIgG-tg and FVB-wt mouse groups, n=6. (**g**) Diagram shows the cross-breeding process using MMTV-IdeS female (scIgG-tg, heterozygous: +/-) and MMTV-PyMT, FVB/N male heterozygous (MMTV-PyMT (+/-) mice. F1 generation pups had four genotypes (scIgG+, PyMT+, scIgG+/PyMT+, and scIgG-/PyMT-). The double positive (scIgG+/PyMT+) and single positive (PyMT+) mice (age matched) were used for spontaneous tumor model study.

Supplementary Figure S3: Detection of lung tumor micro-metastasis in scIgG containing 4T1-IdeS tumor model.

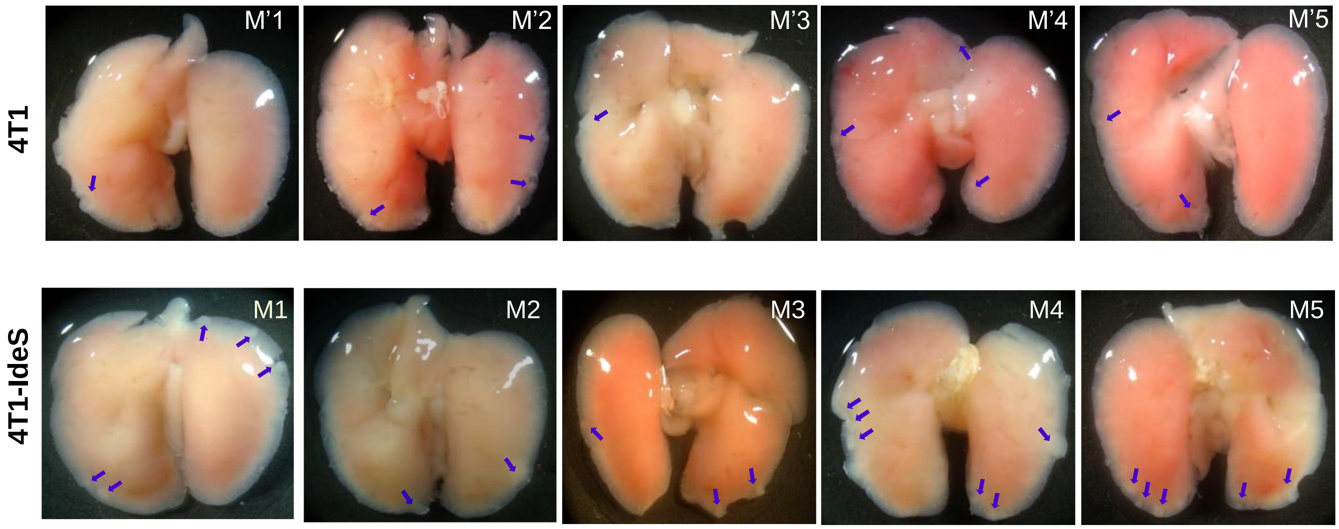

Figure S3. Increased lung micro-metastasis sites were detected in lung tissues from 4T1-IdeS tumor model than that from 4T1 wild type control tumor model, n=5. The 4T1 mice are labelled as M'1, 2, 3, 4, 5 (n=5) and 4T1-IdeS mice are labelled as M1, 2, 3, 4, 5. Blue arrows point to micro-tumor sites (nodules) detected on lung tissues. The images of lung tissue were viewed under a microscope (LEICA S8AP0) with magnifications at 10X and individual tumor microsites were examined under 40X. Images were captured using a camera system (Sony, Model No. NEX-VG30) connected with a microscope at (20X) magnification.

Supplementary Figure S4: Reduced NK cell infiltration and cytotoxicity in tumors with elevated scIgGs.

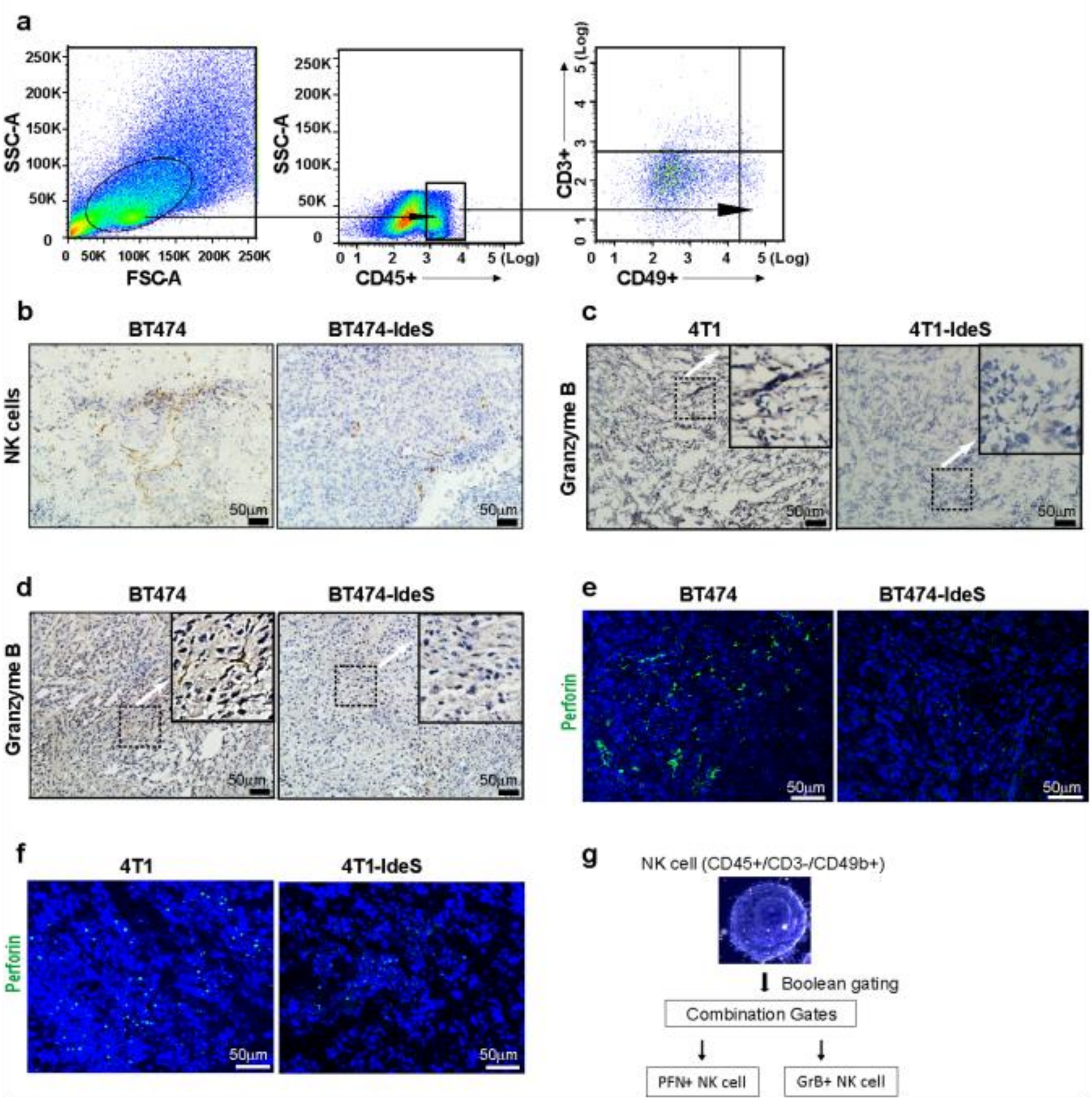

Figure S4. **(a)** Gating strategies used for determination of NK cell population (CD45+/CD49b+/CD3-) in tumor dissociated single cells by flow cytometer. Initial gating used

forward scatter (FSC) and side scatter (SSC) for total living single cells. CD45+ positive cells were gated using anti-mouse CD45-FITC antibody and NK cells were gated in CD45+ immune cell population with CD49+ and CD3- populations. (**b**) NK cells staining in tumor tissues of BT474-IdeS vs BT474 was detected with anti-CD49b antibody using IHC detection. A representative image for each tumor models is shown for each tumor group, 5 images were taken for each tumor slide and each glass slide was made from each mouse tumors. (**c, d**) Granzyme B staining using IHC method and reduced brown staining images were shown in both 4T1-IdeS (**c**) and BT474-IdeS (**d**) tumors. A representative image is shown for each tumor group. Five images were collected per tumor slide and each slide was made from each mouse tumors. (**e, f**) Lower fluorescence staining intensities were detected in scIgG+ tumors in comparison with the control tumors. Perforin expression levels were detected in tumor tissues using IF staining. A representative image from each tumor group is shown for: BT474-IdeS vs BT474 (**e**) and 4T1-IdeS vs 4T1 (**f**). Five images per tumor slides were captured for quantification of staining intensities in the IF images using image j software according to instruction provided for the software. (**g**) Boolean combination gating method used for gating granzyme B (GrB+) and perforin (PFN+) expressing NK cell populations using FlowJo software. To create Boolean combination gates, NK cells (CD49b+/CD3-/CD45+) were gated in the workspace window, then Boolean band “Create Combination Gates” was used for gating subpopulations for GrB+ and PFN+ NK cells.

Supplementary Figure S5: Comparison of CD16 bindings in tumor extracts between scIgG containing tumors and the control tumors.

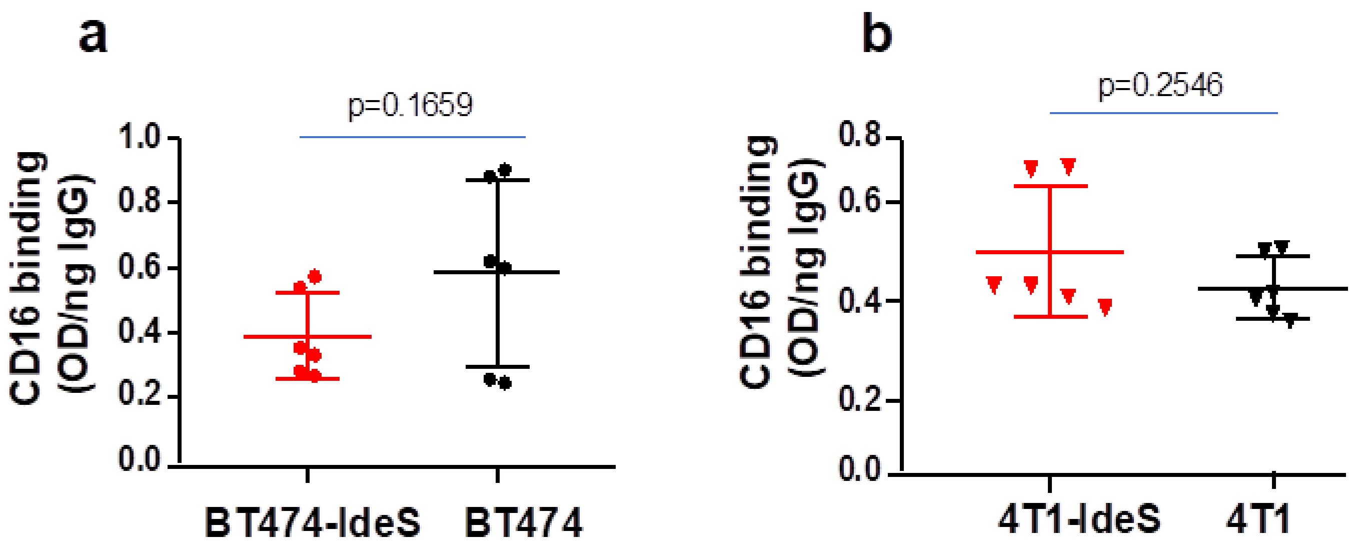

Figure S5. (**a, b**) Levels of CD16 bindings by tumor extracts showed no statistical significance between (**a**) BT474-IdeS vs BT474 tumor lysates and (**b**) between 4T1-IdeS vs 4T1 tumors. Levels of CD16 bindings were normalized with total IgG concentrations in the tumor lysates (OD/ng IgG). Both protein concentrations and total IgG concentrations in tumor lysates were determined using ELISA and used for calculation of CD16 bindings, n=6. Y-axis indicates mean $\pm$ SD.

Supplementary Figure S6: ScIgG containing tumors showed similar low granzyme B and perforin levels in both FcγR knock out and wild type mice.

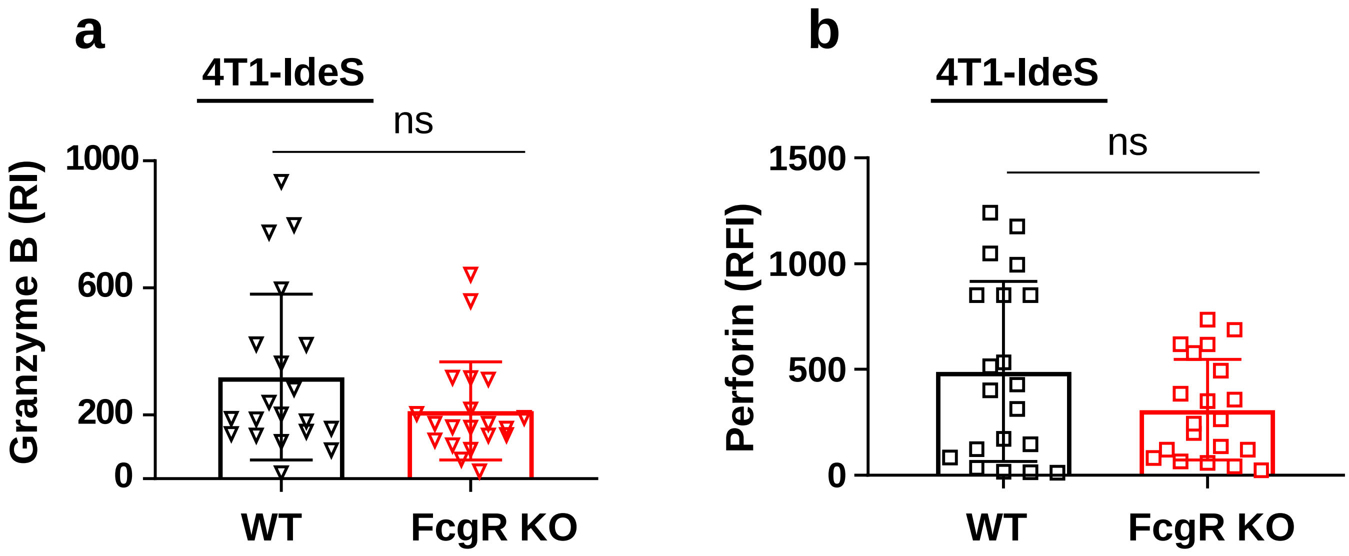

Figure S6. (**a**) 4T1-IdeS tumors from both FcγR knock out (KO) mice and wild type (WT) mice had similarly low levels of granzyme B expression. Staining intensities (RI) were quantitated using image J program and total 20 images (4 images per tumor slides x 5 tumor slides) were used for the quantitation. (**b**) 4T1-IdeS tumors from both FcγR knock out (KO) mice and wild type (WT) mice had similarly low levels of perforin expression. The relative fluorescence intensities (RFI) (Y-axis) were quantified using image J and total 20 images (4 images per tumor slides * 5 tumor slides) were used for the quantitation. Error bars indicate standard deviations (SD), ns for statistically not significant.

Supplementary Figure S7: Proposed working model for AHA targeting scIgGs in mouse tumors.

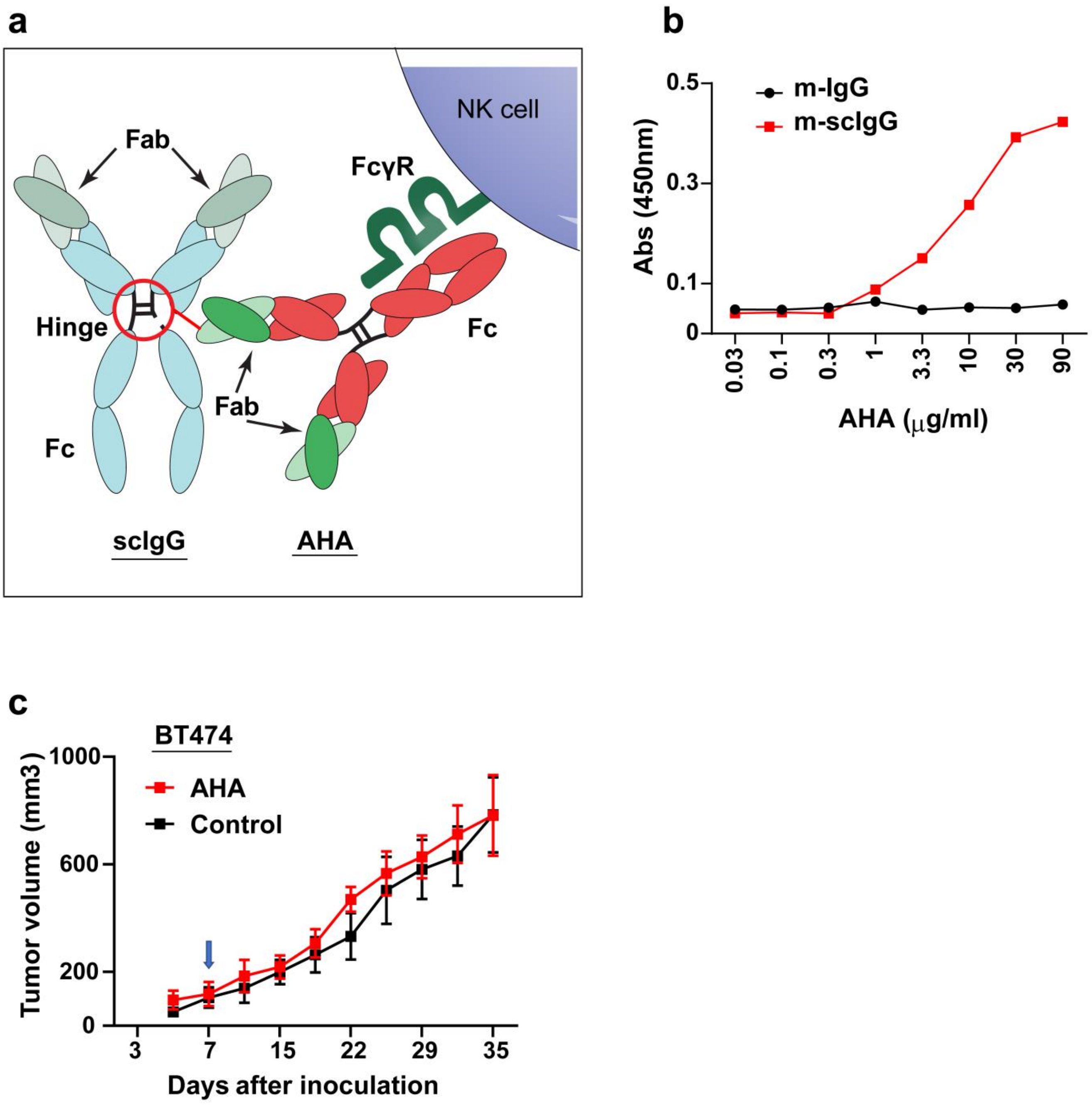

Figure S7. (**a)** Diagram shows a working model for AHA that targets the neoepitope of scIgG (red circle) and provides a functional Fc for engagement of FcγR on NK cells. (**b**) Anti-hinge antibody (AHA) showed specific bindings to mouse scIgGs (m-scIgG) but not the intact mouse

IgG (m-IgG). Mouse IgGs (m-IgG) and m-scIgG (2µg/ml) were coated on high binding 96-well plates and binding of AHA antibody (rabbit IgG) was titrated in a series of concentrations (X-axis). The binding signals of AHA (Y-axis) were detected using a specific anti-rabbit Fc secondary antibody with HRP. (**c**) AHA treatment of BT474 (low scIgG) tumors did not show antitumor efficacy in comparison with isotype antibody (Control). BT474 cancer cells ($5x10^6$ /mouse site) were implanted at mammary sits in nude mice. Mice were treated with either monoclonal AHA or an isotype control IgG antibody at 10 mg/kg for five weekly injections and the blue arrow in the graph indicates the first treatment. The Y-axis shows mean of tumor volume ± SD (standard deviation), n=5.

Supplementary Figure S8: Uncropped immune blot and images used for the supplementary figures.

a

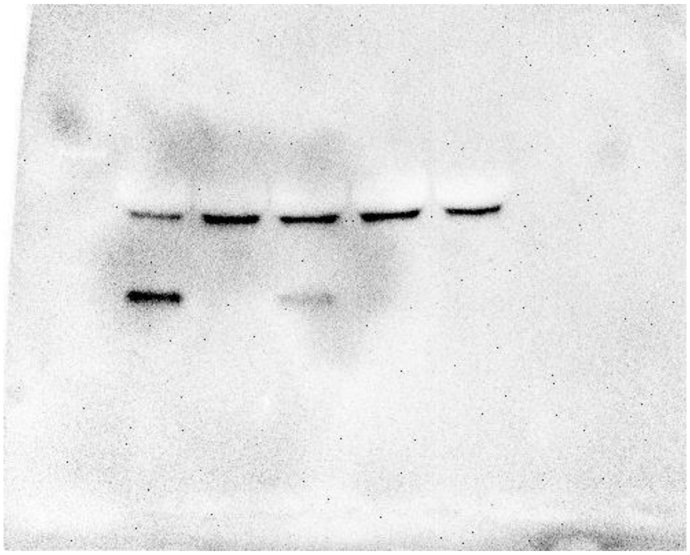

b

Control **scIgG-tg**

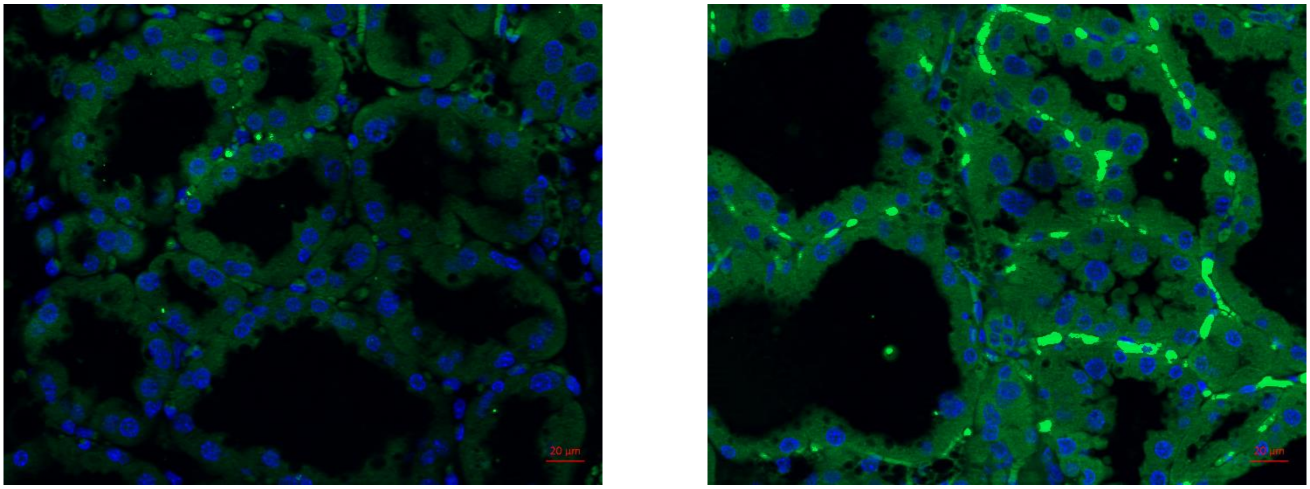

c

scIgG-tg

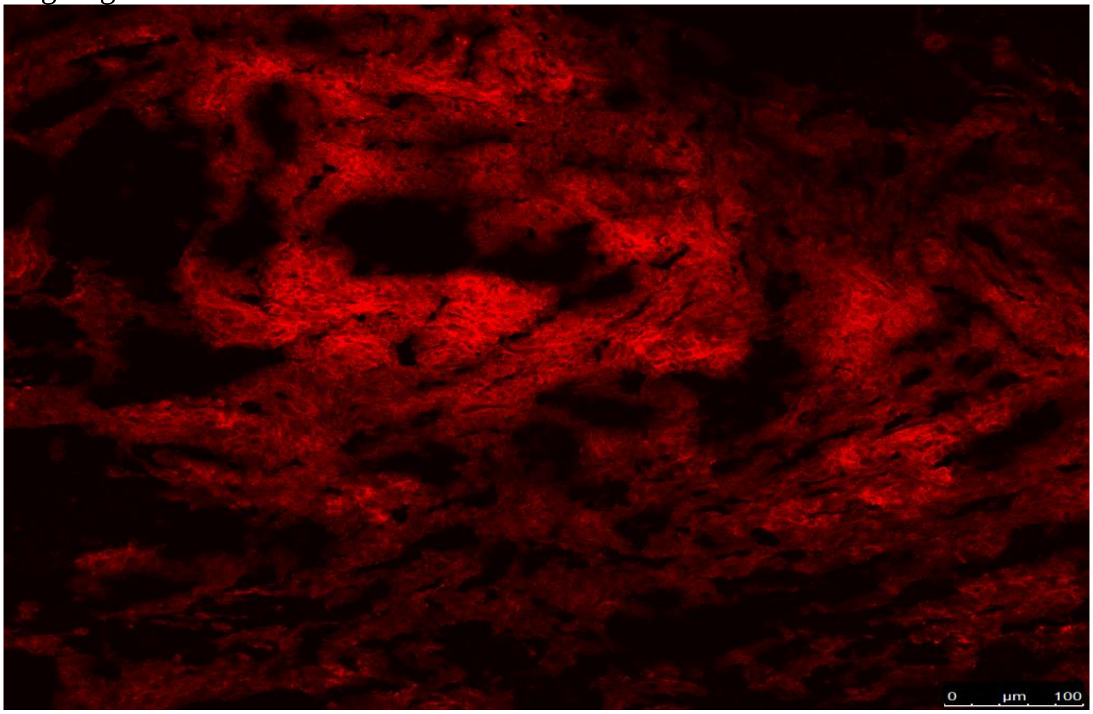

wt

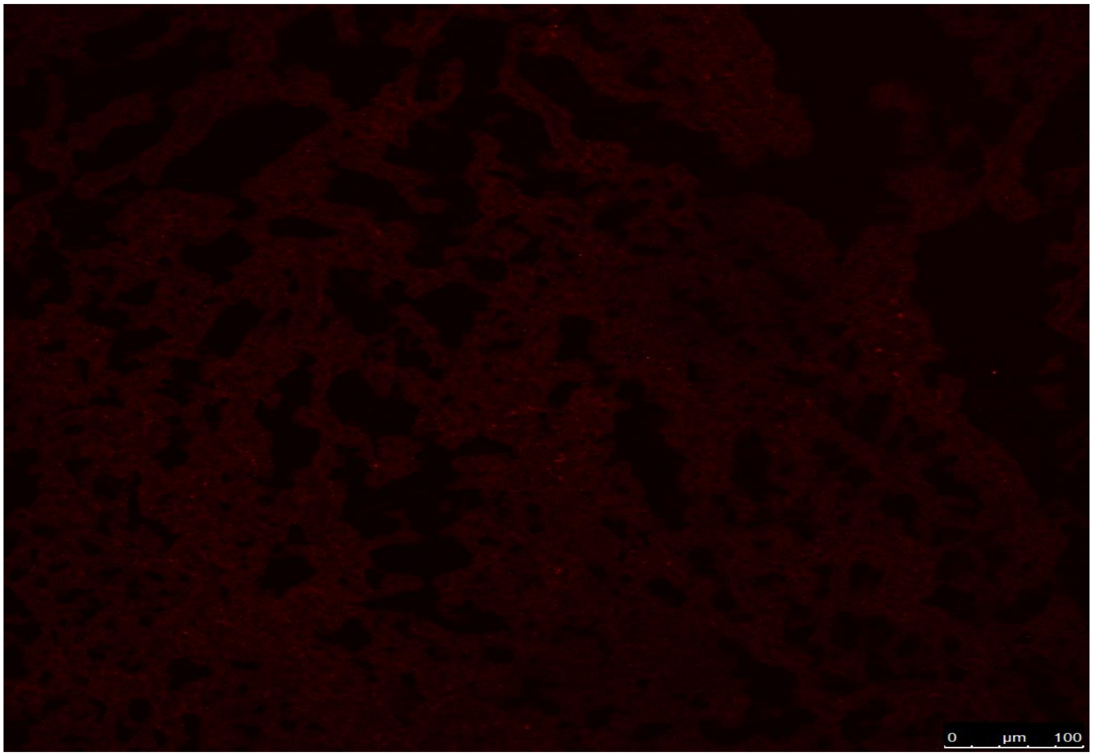

d

BT474-IdeS

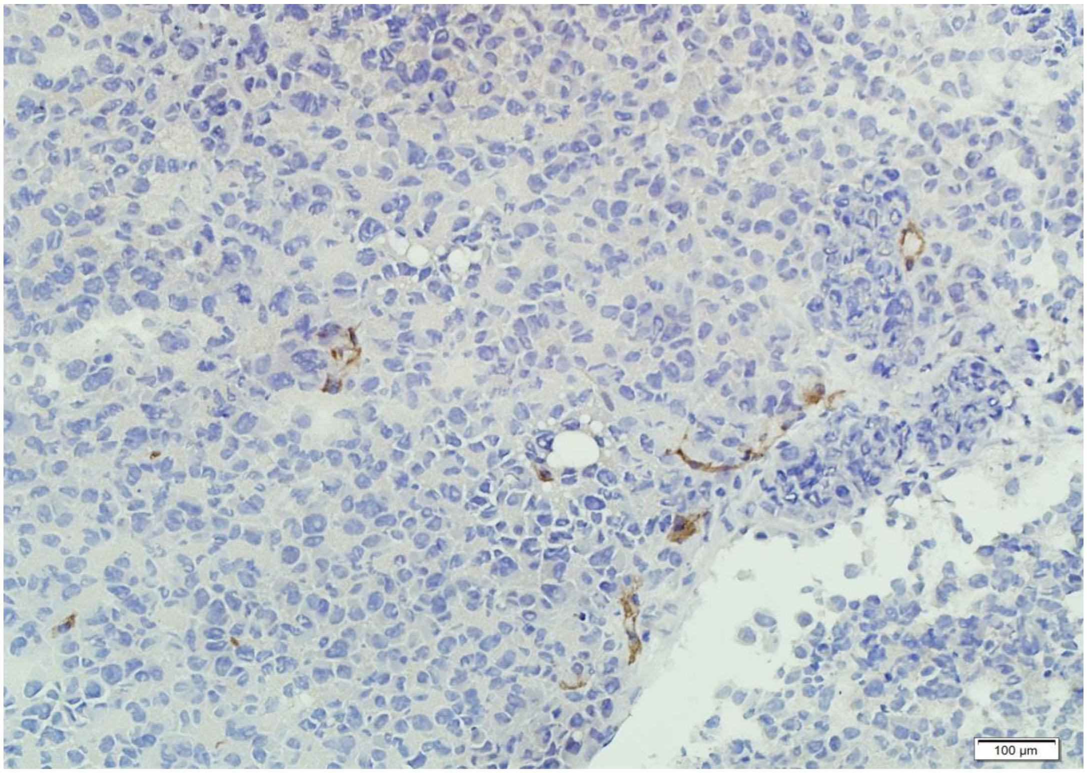

BT474

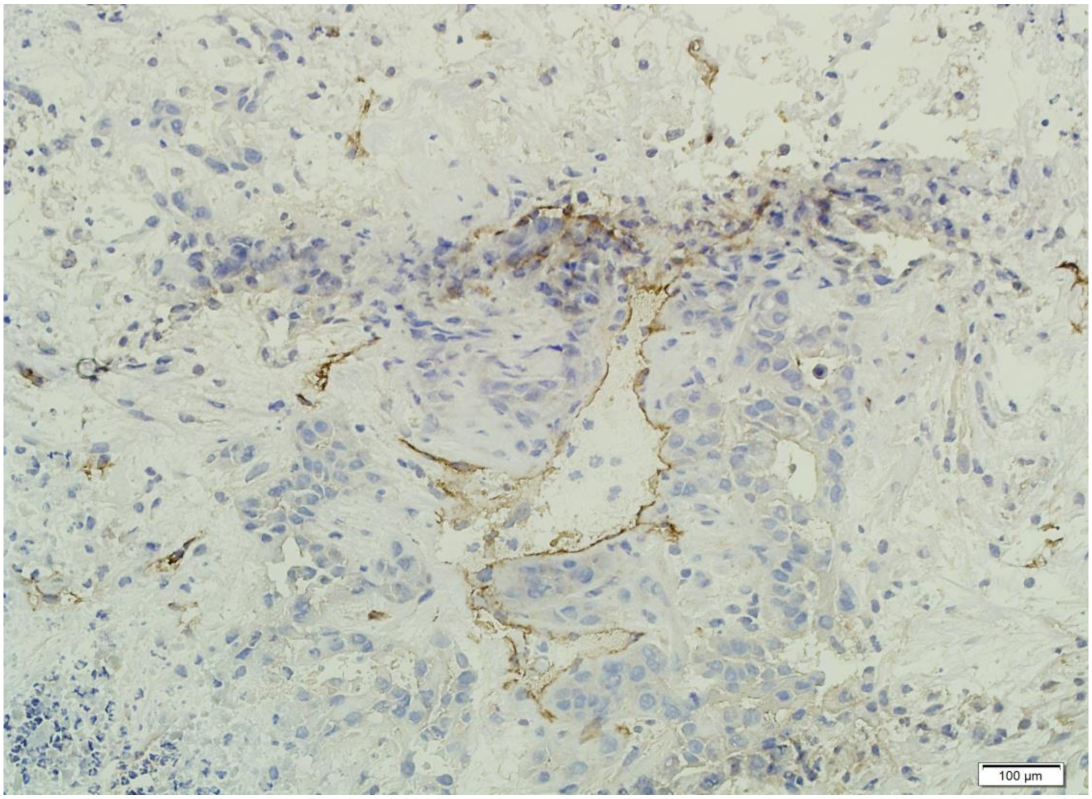

e

4T1-IdeS

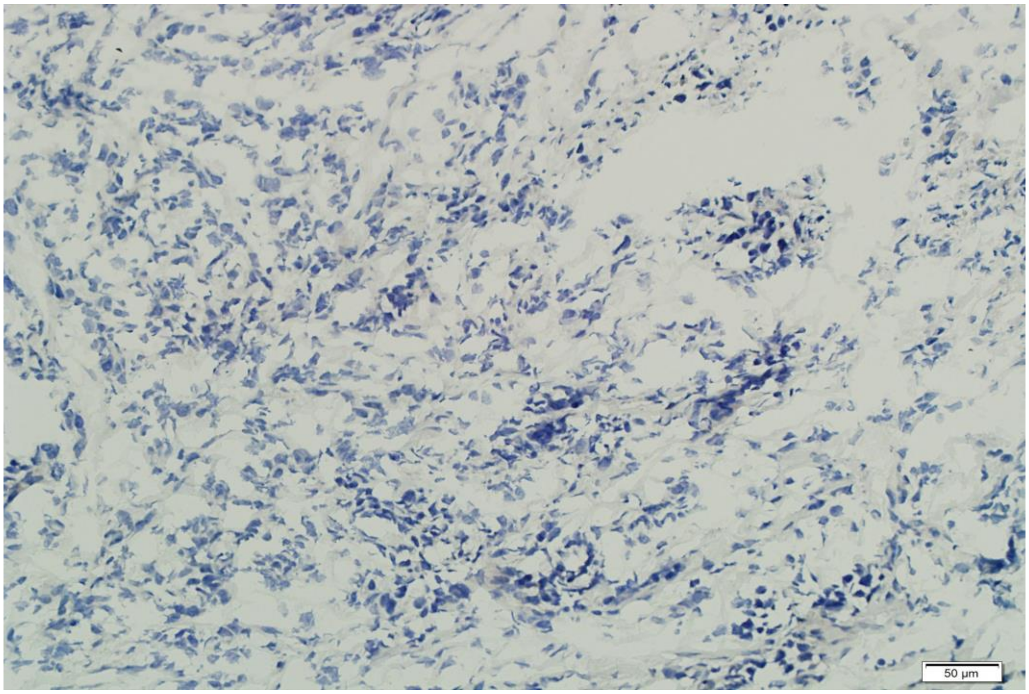

4T1

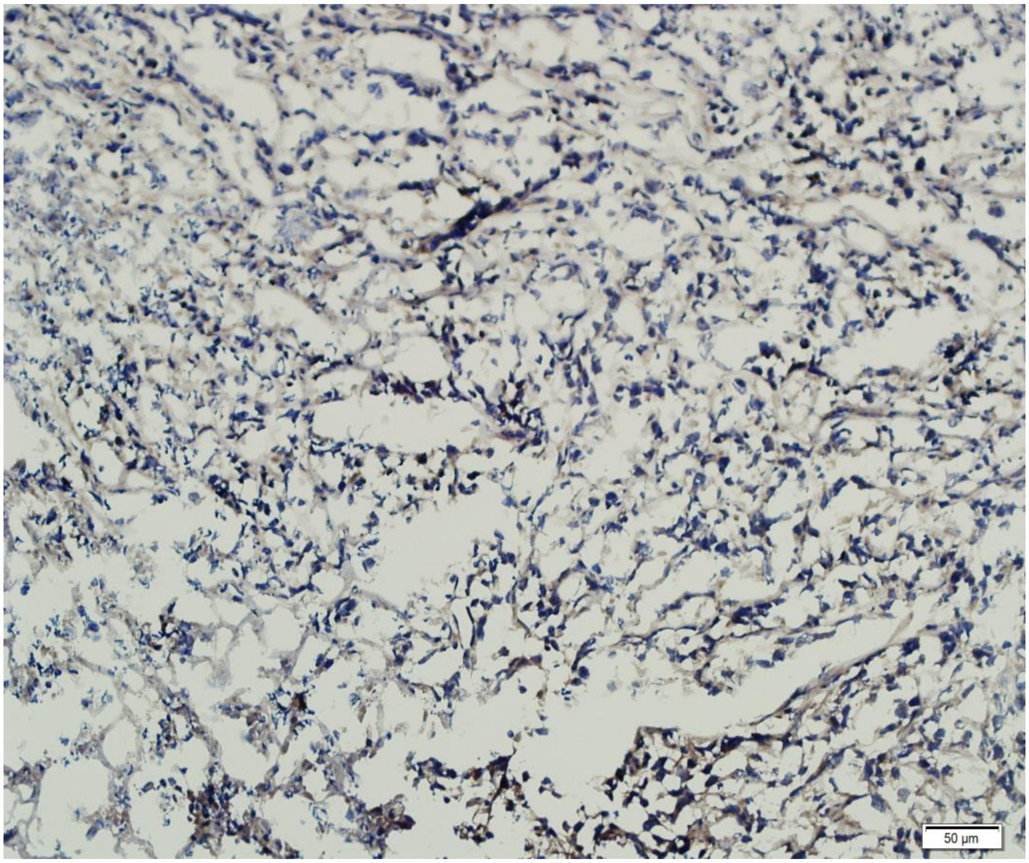
50 µm

f

BT474-IdeS

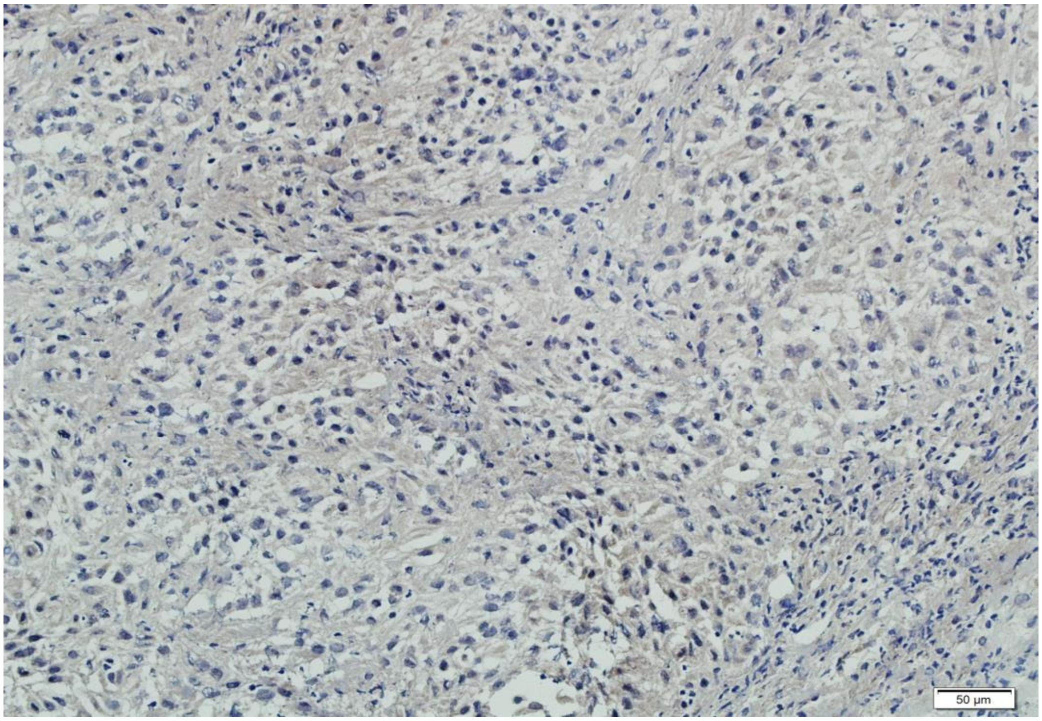

BT474

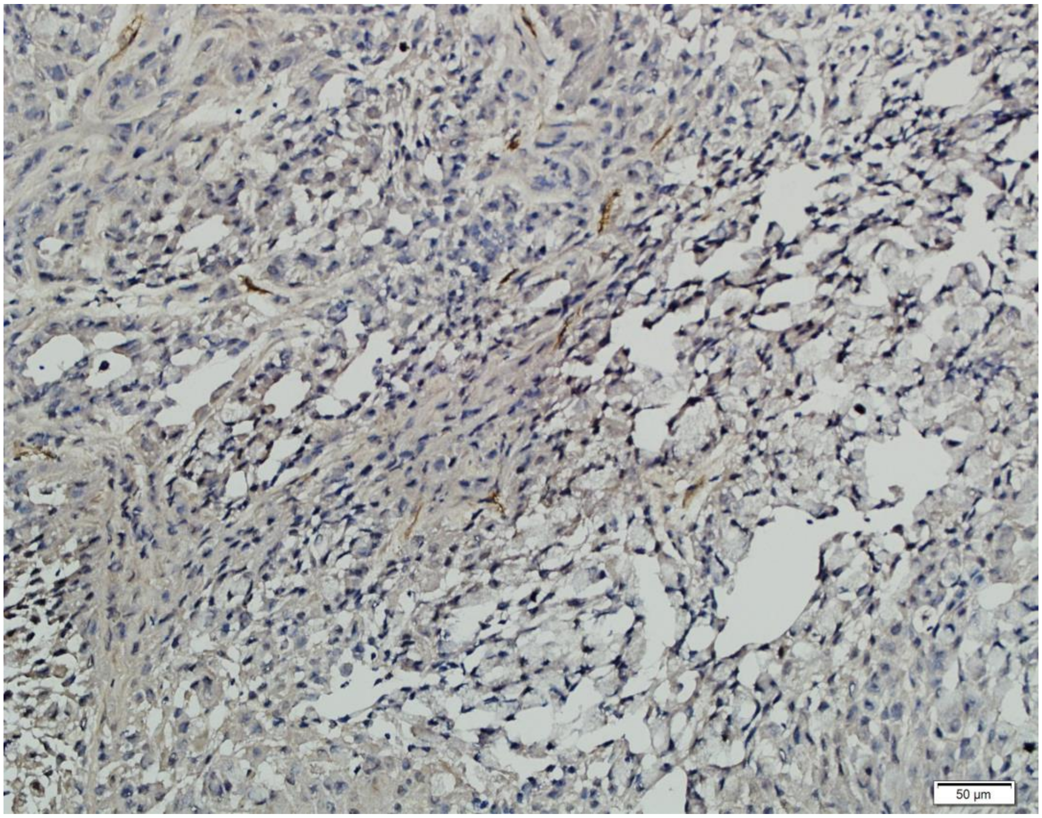

g

BT474-IdeS

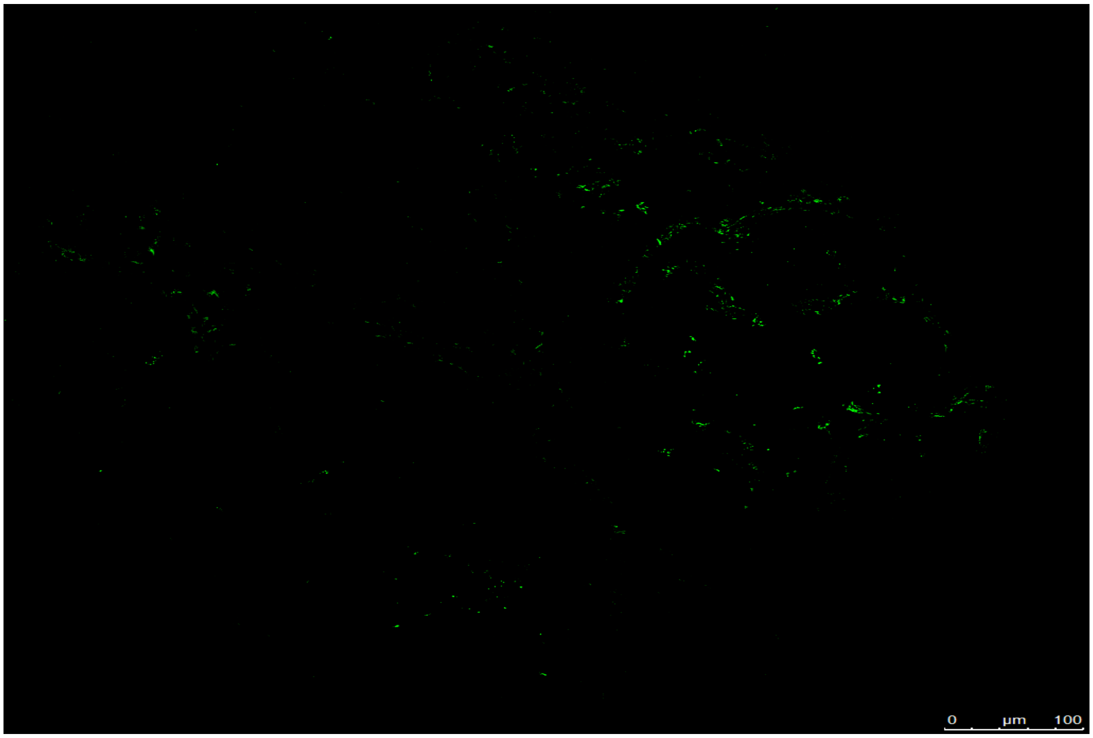

BT474

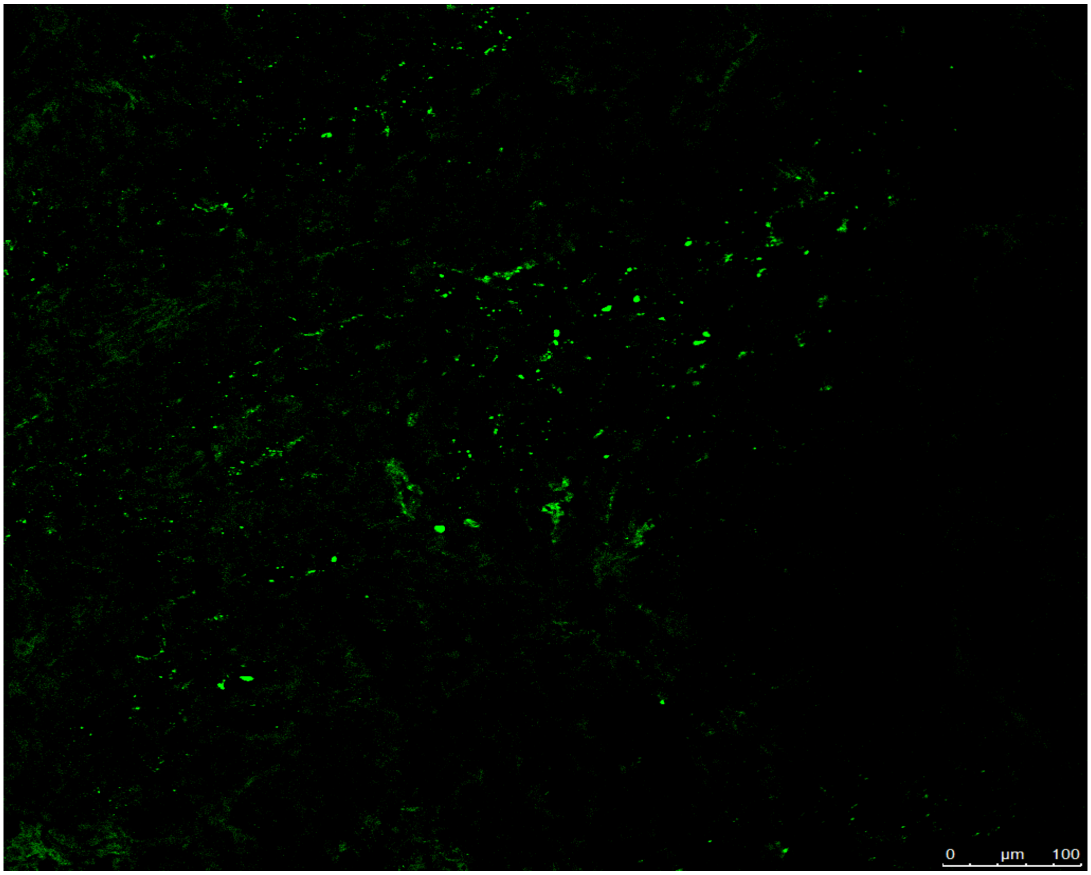

h

4T1-IdeS

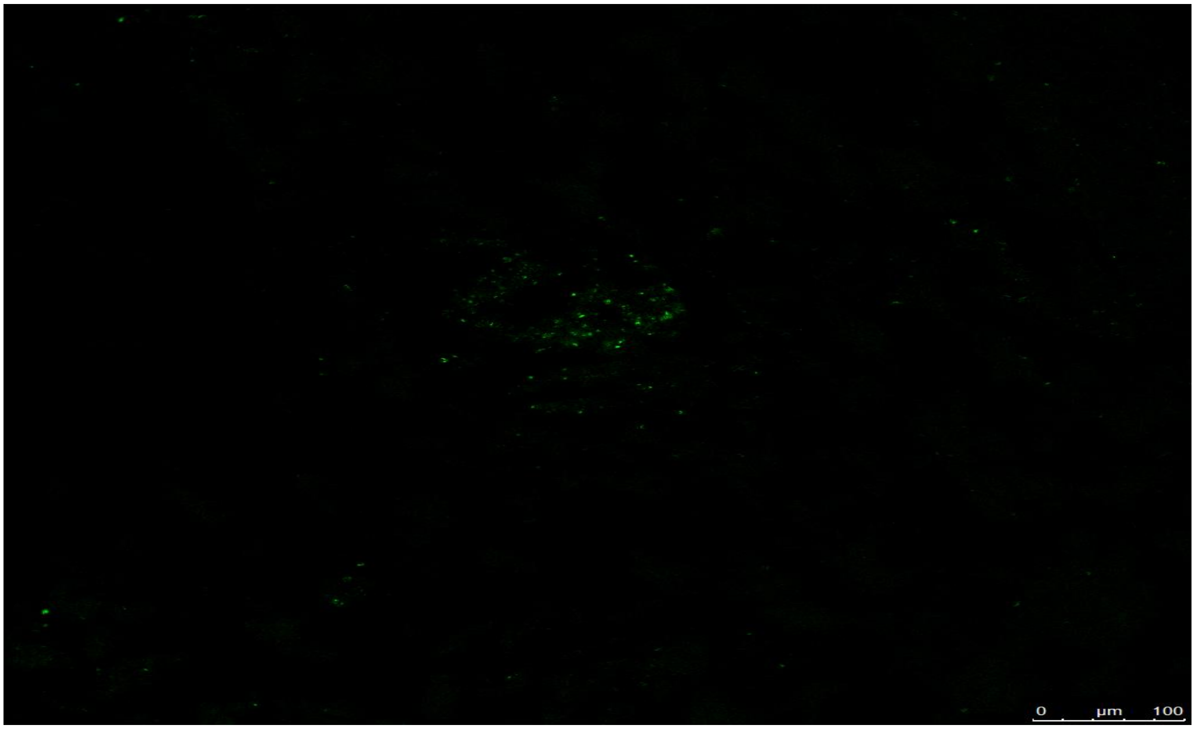

4T1

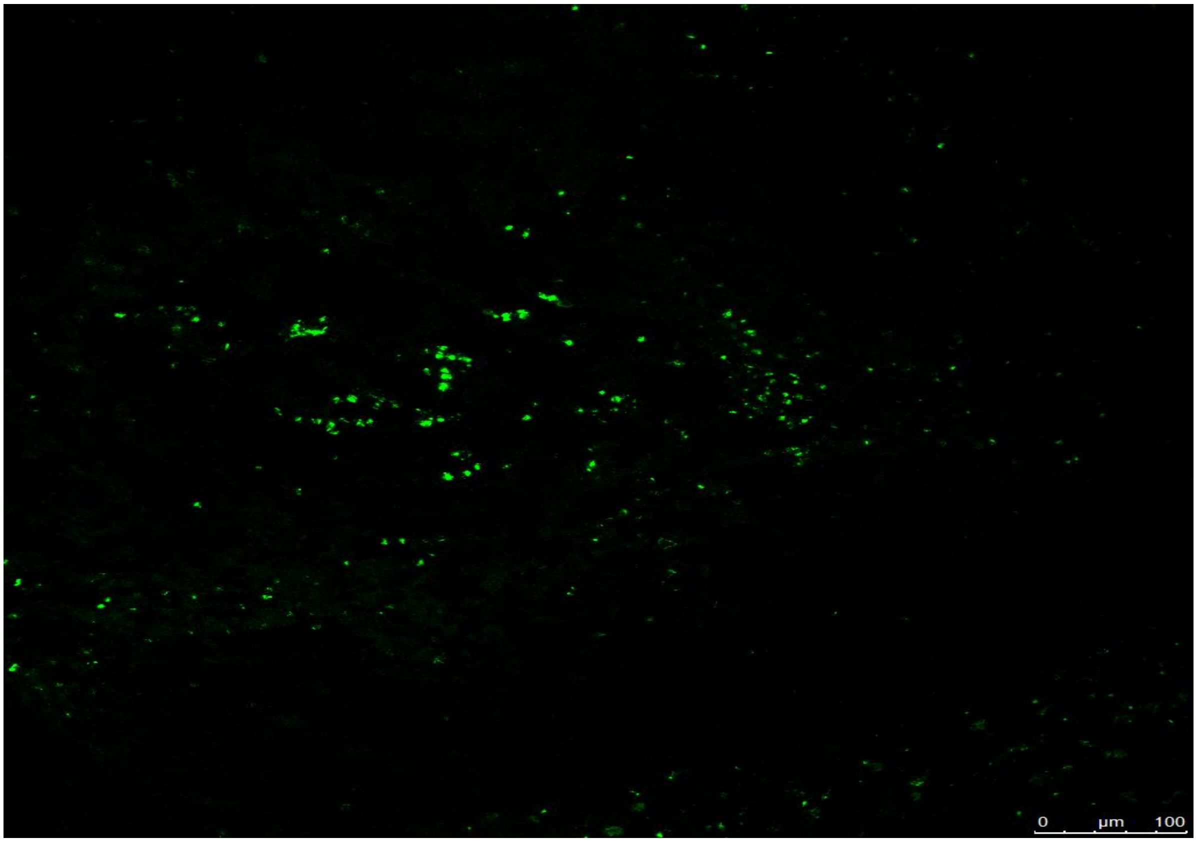

Figure S8. Uncropped immune blot and images used in supplementary information**.**
(a) Uncropped immune blot for ***s***Fig***ure*** 2a. The immune blot was taken using an imager instrument (FluorChem M, Protein***S***imple). From left to right: Lane 1: BT474-IdeS; lane 2: BT474; lane 3: 4T1-IdeS; lane 4: 4T1; lane 5: intact mIgG control. **(b)** Uncropped images for sFigure 2c. **(c)** Uncropped images for sFigure 2d. **(d)** Uncropped images for sFigure 4b. **(e)** Uncropped images for sFigure 4c. **(f)** Uncropped images for sFigure 4d. **(g)** Uncropped images for sFigure 4e. **(h)** Uncropped images for sFigure 4f.
