# Supplementary material for: Impairment of IgG Fc functions promotes tumor progression and suppresses NK cell antitumor actions
Source: Commun Biol. 2022 Sep 14;5:960. doi: 10.1038/s42003-022-03931-7 (PMC9474879; doi:10.1038/s42003-022-03931-7)
Supplement: Supplementary file 3 — Description of Additional Supplementary Files [file 42003_2022_3931_MOESM3_ESM.pdf]

Description of Additional Supplementary Files

File name: Supplementary Data 1
Description: Source data for Figure 1.

File name: Supplementary Data 2
Description: Source data for Figure 2.

File name: Supplementary Data 3
Description: Source data for Figure 3.

File name: Supplementary Data 4
Description: Source data for Figure 4.

File name: Supplementary Data 5
Description: Source data for Figure 5.

File name: Supplementary Data 6
Description: Source data for Figure 6.

File name: Supplementary Data 7
Description: Source data for Figure 7.
