# Supplementary material for: Impairment of IgG Fc functions promotes tumor progression and suppresses NK cell antitumor actions
Source: Commun Biol. 2022 Sep 14;5:960. doi: 10.1038/s42003-022-03931-7 (PMC9474879; doi:10.1038/s42003-022-03931-7)
Supplement: Supplementary file 11 — Reporting Summary [file 42003_2022_3931_MOESM11_ESM.pdf]

nature portfolio

Corresponding author(s): DBPR, CoMMSBIO-21-2681B

Last updated by author(s): 6 July, 2022

Reporting Summary

Nature Portfolio wishes to improve the reproducibility of the work that we publish. This form provides structure for consistency and transparency in reporting. For further information on Nature Portfolio policies, see our Editorial Policies and the Editorial Policy Checklist.

Statistics

For all statistical analyses, confirm that the following items are present in the figure legend, table legend, main text, or Methods section.

n/a	Confirmed	
☐	☒	The exact sample size (n) for each experimental group/condition, given as a discrete number and unit of measurement
☐	☒	A statement on whether measurements were taken from distinct samples or whether the same sample was measured repeatedly
☐	☒	The statistical test(s) used AND whether they are one- or two-sided<br>*Only common tests should be described solely by name; describe more complex techniques in the Methods section.*
☐	☒	A description of all covariates tested
☐	☒	A description of any assumptions or corrections, such as tests of normality and adjustment for multiple comparisons
☐	☒	A full description of the statistical parameters including central tendency (e.g. means) or other basic estimates (e.g. regression coefficient) AND variation (e.g. standard deviation) or associated estimates of uncertainty (e.g. confidence intervals)
☒	☐	For null hypothesis testing, the test statistic (e.g. F, t, r) with confidence intervals, effect sizes, degrees of freedom and P value noted<br>*Give P values as exact values whenever suitable.*
☒	☐	For Bayesian analysis, information on the choice of priors and Markov chain Monte Carlo settings
☒	☐	For hierarchical and complex designs, identification of the appropriate level for tests and full reporting of outcomes
☐	☒	Estimates of effect sizes (e.g. Cohen's d, Pearson's r), indicating how they were calculated

Our web collection on statistics for biologists contains articles on many of the points above.

Software and code

Policy information about availability of computer code

Data collection	IVIS Lumina II in vivo imaging system (IVIS, PerkinElmer), Microscope (Leica S8AP0)/a SONY Camera (Model No. NEX-VG30), Confocal Microscopy (Leica System), gentleMACS Dissociator (Miltenyi Biotec Inc.), A plate reader (SpectraMax M4, Molecular Devices), RayBio's RPPA scanner, Guava easyCyte HT instrument (Millipore), BD FACSCalibur, Olympus DP72 with microscopy.
Data analysis	GraphPad Prism 8.0., Kaplan-Meier survival curves, RayBio® Software, LAS AF Lite (Leica Micro Systems) software, FlowJo software (v10.7.1), ImageJ, Cellsens Standard software, Motic EasyScanner.

For manuscripts utilizing custom algorithms or software that are central to the research but not yet described in published literature, software must be made available to editors and reviewers. We strongly encourage code deposition in a community repository (e.g. GitHub). See the Nature Portfolio guidelines for submitting code & software for further information.

Data

Policy information about availability of data

All manuscripts must include a data availability statement. This statement should provide the following information, where applicable:

- Accession codes, unique identifiers, or web links for publicly available datasets
- A description of any restrictions on data availability
- For clinical datasets or third party data, please ensure that the statement adheres to our policy

Patient clinical characteristics data are provided in S1 table; data used to make graphs are available in Excell data sheet and/or Graphpad file. All images taken from Confocal or light microscope or image scanner are available and saved in JPEG or TIFF files.

Field-specific reporting

Please select the one below that is the best fit for your research. If you are not sure, read the appropriate sections before making your selection.

☒ Life sciences ☐ Behavioural & social sciences ☐ Ecological, evolutionary & environmental sciences

For a reference copy of the document with all sections, see nature.com/documents/nr-reporting-summary-flat.pdf

Life sciences study design

All studies must disclose on these points even when the disclosure is negative.

Sample size	For animal studies: we used from 5 mice to more than 20 mice based on study design and publications. Sample sizes are indicated in Figure legends of individual graphs.
Data exclusions	N/A
Replication	All cell culture studies and in vitro assays were repeated at least two times
Randomization	Mice with tumor implantations for treatment study were randomly grouped before starting treatment and cancer cells were prepared as one pool for in vivo injections.
Blinding	Antibody preparation and in vivo study were conducted by different investigators to reduce subjective effects on observation in tumor treatment study.

Reporting for specific materials, systems and methods

We require information from authors about some types of materials, experimental systems and methods used in many studies. Here, indicate whether each material, system or method listed is relevant to your study. If you are not sure if a list item applies to your research, read the appropriate section before selecting a response.

Materials & experimental systems

n/a	Involved in the study	
☐	☒	Antibodies
☐	☒	Eukaryotic cell lines
☒	☐	Palaeontology and archaeology
☐	☒	Animals and other organisms
☒	☐	Human research participants
☐	☒	Clinical data
☒	☐	Dual use research of concern

Methods

n/a	Involved in the study	
☒	☐	ChIP-seq
☐	☒	Flow cytometry
☒	☐	MRI-based neuroimaging

Antibodies

Antibodies used	Anti-mouse CD49b (for NK cells) (Invitrogen, 14-5971-85), anti-mouse granzyme B (Abcam, ab4059), anti-mouse perforin monoclonal antibody (eBioOMAK-D), FITC-conjugated anti-Mouse CD45 [BD Pharmingen, 5553079)], PerCP-CyTM5.5 anti-Mouse CD3 [BD Pharmingen, 560527] and PE-conjugated anti-Mouse CD49b [BD Pharmingen, 561066], goat anti-mouse IgG specific secondary antibody with HRP conjugates (Jackson ImmunoResearch), goat anti-mouse IgG-Fc-HRP conjugate (1:4000) (Jackson Immune Research Laboratory, PA). An optimized mixture of three specific anti-hinge polyclonal antibodies (Ref. 14), biotinylated anti-rabbit/mouse antibody (Kit from Vector lab), biotinylated anti-rat antibody IgG (Vector lab, BA-4001).
Validation	Antibodies purchased from commercial sources are based on information from vendors. In-house produced antibodies were validated using commonly used methods for purity, specificity, and binding affinities.

Eukaryotic cell lines

Policy information about cell lines

Cell line source(s)	BT474 and 4T1 cells were obtained from ATCC. The Met1 cancer cell line was a gift from Dr. Philipp Scherer's laboratory at University of Texas Southwestern Medical Center (UTSW), Dallas, Texas. BT474-IdeS, and 4T1-IdeS stable cell lines were constructed as we described previously (11, 13) using the RevTet-off system (Clontech Laboratories).
Authentication	Cell lines were purchased from ATCC .
Mycoplasma contamination	No observed contamination during the study.

Commonly misidentified lines
(See ICLAC register)

No.

Animals and other organisms

Policy information about studies involving animals; ARRIVE guidelines recommended for reporting animal research

Laboratory animals	ATHYMIC NUDE NU/NU, Female, 8 weeks, BALB/c, Female, 8 weeks, FVB, Female, 8 weeks, C.129P2(B6)-Fcer1gtm1Rav N12, Female, 8 weeks, Crossed FVB-IdeS with FVB/MMTV-PyVT, Female, 8 week, FvB/PyvT-mmtv (Tg) and Crossed with FVB-IdeS.
Wild animals	N/A.
Field-collected samples	N/A.
Ethics oversight	Animal protocol number: AWC-19-0051 and the protocol was approved by the Animal Welfare Committee of the University of Texas Medical School at Houston.

Note that full information on the approval of the study protocol must also be provided in the manuscript.

Clinical data

Policy information about clinical studies
All manuscripts should comply with the ICMJE guidelines for publication of clinical research and a completed CONSORT checklist must be included with all submissions.

Clinical trial registration	Not applicable
Study protocol	TMA slides were made in accordance with a clinical protocol (HREC/12/LPOOL/158; Project No: 12/092) approved by the South Western Sydney Local Health District Ethics Executive Committee.
Data collection	Data was de-identified before used for retrospective analysis.
Outcomes	Retrospective study

Flow Cytometry

Plots

Confirm that:

☒ The axis labels state the marker and fluorochrome used (e.g. CD4-FITC).

☒ The axis scales are clearly visible. Include numbers along axes only for bottom left plot of group (a 'group' is an analysis of identical markers).

☒ All plots are contour plots with outliers or pseudocolor plots.

☒ A numerical value for number of cells or percentage (with statistics) is provided.

Methodology

Sample preparation	The mouse tumors were dissociated to single cells with the gentleMACS C tube by gentleMACS Dissociator (Miltenyi Biotec)
Instrument	Guava easyCyte HT instrument (Millipore) and FACSCalibur
Software	FlowJo-v10.7.1 and 7.6.5
Cell population abundance	All of the gates were established based on creation of combinations of the negative control and single staining from each antibody.
Gating strategy	Initial gating was on all lymphocytes as approximated by forward scatter (FSC) and side scatter (SSC), then doublets were eliminated. Next, live CD45+ positive cells were detected by using anti-mouse CD45-FITC antibody which is one of the most common gating strategy used in flow cytometry analysis. The selected live CD45+ cells with mixture staining tube, then from CD3- cells and NK+ cells were gated to obtained the percentage of expression level.

☒ Tick this box to confirm that a figure exemplifying the gating strategy is provided in the Supplementary Information.
